# Health providers’ and pregnant women’s perspectives about smoking cessation support: a COM-B analysis of a global systematic review of qualitative studies

**DOI:** 10.1186/s12884-021-03773-x

**Published:** 2021-08-12

**Authors:** Ratika Kumar, Leah Stevenson, Judith Jobling, Yael Bar-Zeev, Parivash Eftekhari, Gillian S. Gould

**Affiliations:** 1 School of Medicine and Public Health, The University of Newcastle, University Dr, Callaghan, New South Wales 2308 Australia; 2 Braun School of Public Health and Community Medicine Hebrew University - Hadassah Medical Center, PO Box 12272, Jerusalem, 91120 Israel

**Keywords:** Pregnant women, Health professionals, Smoking cessation, Health services, Qualitative, COM-B

## Abstract

**Background:**

Smoking cessation in pregnancy has unique challenges. Health providers (HP) may need support to successfully implement smoking cessation care (SCC) for pregnant women (PW). We aimed to synthesize qualitative data about views of HPs and PW on SCC during pregnancy using COM-B (Capability, Opportunity, Motivation, Behaviour) framework.

**Methods:**

A systematic search of online databases (MEDLINE, EMBASE, PsycINFO and CINAHL) using PRISMA guidelines. PW’s and HPs’ quotes, as well as the authors’ analysis, were extracted and double-coded (30%) using the COM-B framework.

**Results:**

Thirty-two studies included research from 5 continents: twelve on HPs’ perspectives, 16 on PW’s perspectives, four papers included both. HPs’ capability and motivation were affected by role confusion and a lack of training, time, and resources to provide interventions. HPs acknowledged that advice should be delivered while taking women’s psychological state (capability) and stressors into consideration. Pregnant women’s physical capabilities to quit (e.g., increased metabolism of nicotine and dependence) was seldom addressed due to uncertainty about nicotine replacement therapy (NRT) use in pregnancy. Improving women’s motivation to quit depended on explaining the risks of smoking versus the safety of quit methods. Women considered advice from HPs during antenatal visits as effective, if accompanied by resources, peer support, feedback, and encouragement.

**Conclusions:**

HPs found it challenging to provide effective SCC due to lack of training, time, and role confusion. The inability to address psychological stress in women and inadequate use of pharmacotherapy were additional barriers. These findings could aid in designing training programs that address HPs’ and PW’s attitudes and supportive campaigns for pregnant smokers.

**Supplementary Information:**

The online version contains supplementary material available at 10.1186/s12884-021-03773-x.

## Background

Smoking tobacco during pregnancy is an established risk factor for a range of health problems for mothers and the baby, including long term complications in childhood. Exposure to tobacco smoke in-utero increases the risk of still birth, low birth weight and small for gestational age babies [1]. Women who smoke during pregnancy are more likely to experience obstetric complications such as spontaneous abortions, placental abruption, placenta previa, premature labour, and ectopic pregnancies compared to women who don’t smoke during pregnancy [1]. Globally, 1.7% of pregnant women (PW) smoke during pregnancy [2]. Five countries namely Ireland, Uruguay, Bulgaria, Spain and Denmark have the highest rates of smoking in pregnancy ranging from 25 to 38%. Research suggests that a significant proportion of women stop smoking when they become pregnant, mainly to safeguard their baby from the harms of smoking [3]. However, worldwide, 50% of women who smoke continue to do so during pregnancy [2]. Experiencing social or economic disadvantage, particularly poverty, living in a normalized smoking environment, low access to healthcare and a highly stressful life are significant predictors of smoking during pregnancy [4]. For example, Indigenous women from developed countries, who may be exposed to all of the aforesaid, have double the smoking prevalence compared to their pregnant, non-Indigenous counterparts [5]. Other risk factors for smoking during pregnancy include being an older mother, teenage mother, multiparty, high nicotine dependence, experiencing intimate partner violence or mental health issues such as depression [4].

### The health provider’s role in smoking cessation

High quality evidence suggests behavioral interventions such as counseling, feedback, and incentives increase smoking cessation rates in pregnancy [6]. Pragmatic research also suggests that nicotine replacement therapy (NRT) increases smoking cessation rates among PW, although more research to account for the higher nicotine metabolism during pregnancy and research on higher doses of NRT are needed [7]. Smoking cessation guidelines from Australia, New Zealand, Canada, and the United Kingdom recommend NRT use by PW to achieve smoking cessation if behavioral methods are not successful [7]. The onus of translating the existing smoking cessation research lies on health providers (HPs) who remain the mainstay for providing smoking cessation care to PW. HPs that care for PW come from several disciplines, including medical practitioners, midwives, and Community Health Workers. Clinical smoking cessation guidelines usually recommend a structured approach such as the 5As (Ask, Assess, Advise, Assist and Arrange) or the ABC (Ask, Brief advice and Cessation support) [8] to HPs to ensure the completeness of the smoking cessation intervention. Despite these guidelines, smoking cessation interventions may be underutilized by HPs [9–11]. Additionally, there is little practical guidance for HPs such as how to weigh up the risks versus benefits of using NRT in pregnancy, and how to titrate the dosage of NRT to account for PW’s faster metabolism [7]. There is limited research about HPs’ knowledge and attitudes about providing smoking cessation care to smoking PW during their consultations [12, 13]. Given that smoking cessation in pregnancy has unique challenges, HPs may need additional training and skills for the successful implementation of smoking cessation interventions for PW.

**Fig. 1:**
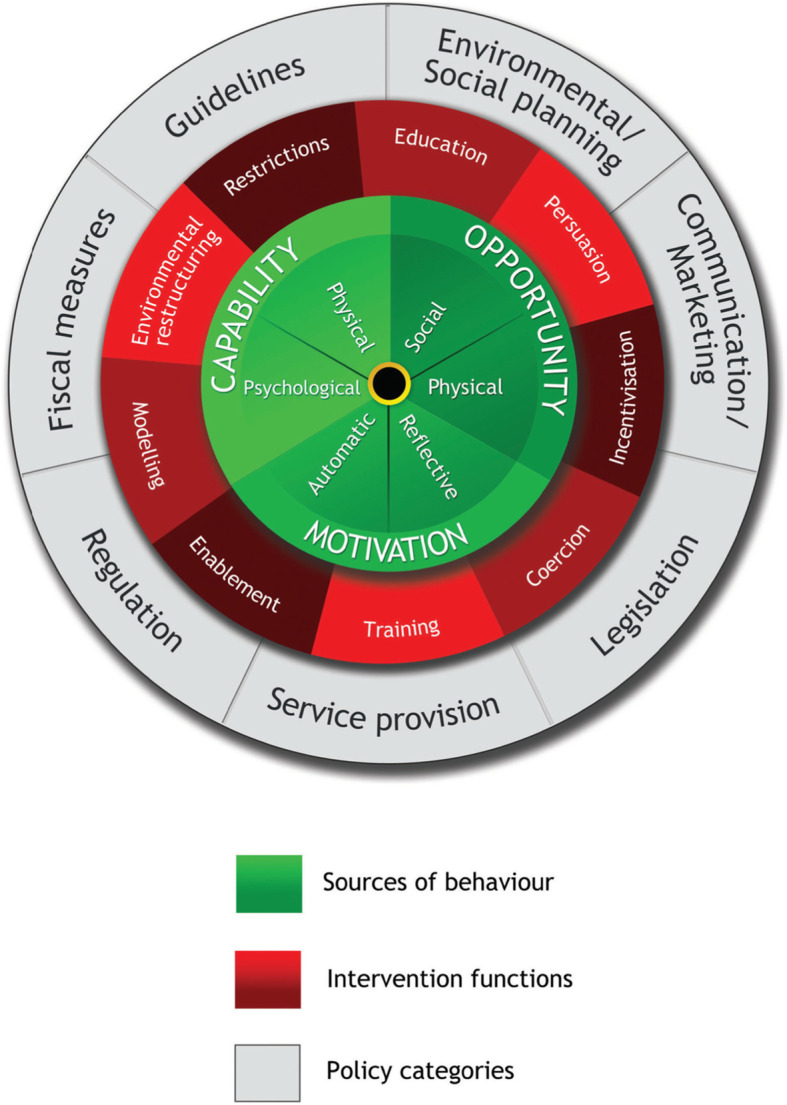
Behaviour Change Wheel (BCW) (Reproduced with permissions from authors)

A large number of theories and models have been proposed to explain the complex science of behavior change. The Behaviour Change Wheel (BCW) is a parsimonious model that takes into account multiple behavior change theories (See Fig. 1). At its center (green hub) is the COM-B model [14]. COM-B is an acronym for capability (C)- physical and psychological, Opportunity (O)- physical and social and Motivation (M)- automatic and reflective, all of which drive behavior change (B) [14]. Most behavior change interventions incorporate one or more of these behavior change principles. However, a successful intervention ideally should take all three tiers of the wheel into consideration [15]. Consequently, the COM-B and BCW can be used to analyze a behavior change intervention to identify if an intervention has used a systematic approach to achieve its desired outcomes, what mechanism of actions were operationalized and if the intervention failed, the possible reasons for its failure [15]. Intervention functions and policies are captured in the middle (red) and outer (grey) rings respectively.

This systematic review aims to synthesize published qualitative research about smoking cessation care provided by HPs to PW who smoke. The primary objective was to explore both HPs’ and PW’s perspectives on smoking cessation care using a COM-B framework with reference also to the BCW. A secondary aim was to identify intervention functions how the capability, opportunity, and motivation of HPs could be improved to provide optimum smoking cessation care during pregnancy. Intervention functions are broad categories of means by which an intervention can change behaviour. Intervention functions as described by Michie S [15] include education, persuasion, incentivization, coercion, training, restriction, environmental restructuring, modelling and enablement. We have highlighted intervention functions (IF) where they were apparent from the extracted data, but not for every finding.

## Methods

### Data sources and study selection

**Fig. 2:**
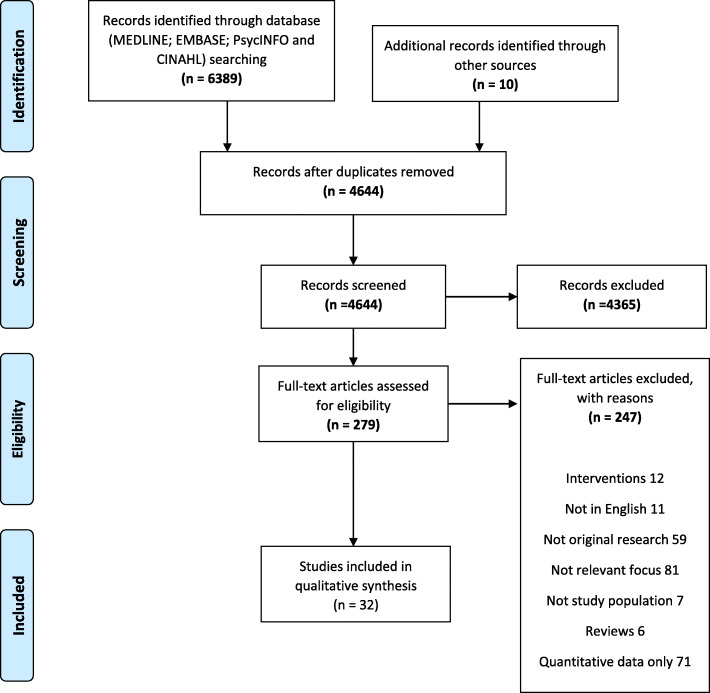
Summary of the article selection process as recommended by the PRISMA statement

Underpinned by PRISMA guidelines, a systematic search was carried out in research databases including MEDLINE; EMBASE; PsycINFO and CINAHL until March 2020 (see Fig. 2). Keywords and Medical Subject Headings (MESH) terms related to ‘Health providers,’ Search terms: “Attitudes and practices; “smoking” and; “pregnancy” were used (Supplemental file 1: Full search strategy in Medline).

#### Inclusion criteria

Peer-reviewed, qualitative, or mixed methods studies, written in English, were included. The topics explored were: the views of any HPs about the provision of established forms of smoking cessation care to PW (for example the 5As or smoking cessation guidelines) in any setting and papers exploring women’s views on the smoking cessation care received from their HPs. There was no restriction on publication date.

#### Exclusions

Studies exclusively reporting quantitative data, evaluating new programs or interventions (papers reporting qualitative data on established practices were not considered new), in non-peer reviewed journals, or only minimally covered the topic.

This review was a part of a larger review registered with PROSPERO in 2015: CRD42015029989. The quantitative data were analyzed and published separately [11].

### Data extraction

The review team included a General Practitioner (GG), Public Health Physician (YBZ), Public Health Dentist (RK), Health Promotion Expert (LS), Epidemiologist (JJ), Psychologist (GRG), Gender and Health Expert (PE) and Behavioural Scientist (LT). Authors have an extensive background in tobacco cessation, epidemiology, and qualitative research. Electronic databases were searched by LS, RK, YBZ and LT, who independently screened the titles and abstracts of the retrieved articles. Full-text articles were independently by two authors (YBZ, LT, JJ, GRG, LS, PE). Date range was from August 2015 to March 2020. A third author adjudicated discrepancies by mutual discussion (GG). Reference lists of the included articles were searched for additional papers and original articles thus retrieved included if they fulfilled the eligibility criteria. We only analyzed the study with available results as a part of the published manuscript and did not contact the authors for further detail. Microsoft Excel was used to record the study characteristics namely title, authors, year of publication, setting, aims, study design, methodological orientation, quotes (first-order constructs), themes and subthemes (second-order constructs), explanatory models (third-order constructs), sample size and focus of the interviews. Data were extracted by one author (LS) from the full texts of the articles, quality checked by a second author (YBZ and GRG) for twelve of the articles (45%).

### Analysis

#### Quality assessment

Since there are very few established quality assessment tools for qualitative studies and due to the research team’s prior experience, the Hawker Quality Assessment Tool [16, 17] was used to for the quality assessment of the studies. This tool assesses studies using nine questions; potential ratings and corresponding numerical scores were good (4), fair (3), poor (2), and very poor (1). Scores for each study were totaled to give a score indicative of the overall quality of the study, ranging from a minimum of 9 points to a maximum of 36 points. Studies were classified as high quality (30–36 points); medium quality (24–29 points) or low quality (9–24 points) [17]. Initially 5% of randomly selected papers were assessed by LS, YBZ and GB. Where the reviewers assigned different domain scores to a study, the differences were discussed among co-authors with GG adjudicating to resolve the difference to arrive at a final domain score. The rest of the papers were then scored by LS informed by the discussion and adjudication.

#### COM-B analysis

For this review, the BCW [14, 18] and the COM-B model [14, 18] were used to analyze the smoking cessation care delivered by HPs or received by PW who smoked. We coded for all aspects of care delivery from the viewpoint of HPs and women, including barriers and facilitators as well as the actual care delivered. First-order constructs (quotes) from HPs and PW and second-order constructs (interpretations by authors or themes) were extracted line by line from the included papers and coded using the COM-B framework and its sub-components. Quotes were multi-coded if they reflected more than one component or subcomponent of the COM-B framework. LS completed coding; 30% of the papers were double coded to ensure consistency (JJ and RK). GG reviewed the coding to ensure consistency. BCW Policy categories (grey zone BCW- Fig. 1) and Intervention functions (red zone BCW- Fig. 1) were selected by GG and RK to describe which intervention functions and policy measures (aimed at HPs and/or PW) were indicated by the data, according to the BCW. The data was synthesized by creating a narrative within each theme within the COM-B framework. Coders (JJ, RK and LS) continuously reflected on their own inherent biases. In particular, the researchers were aware during the data collection and analysis process of their backgrounds in smoking cessation and intervention delivery might bias the interpretation of findings. Assumptions while analyzing the data were discussed with GG for arbitration.

## Results

This review included 32 qualitative and mixed methods studies: twelve focused exclusively on the HPs’ perspectives [19–30], 16 exclusively on PW’s perspectives [31–46], while four papers included both [47–50]. Twenty studies were conducted in Europe [19, 21, 22, 25–27, 30, 31, 34, 36, 38, 39, 41–45, 47, 49, 51], eight in the Oceania region [20, 24, 29, 33, 37, 40, 46, 50], two in the USA [23, 35] and one each in South America [48] and Africa [28]. HPs included doctors, nurses, midwives, Aboriginal Health Workers, pharmacists and other allied HPs. Please see Supplementary file 2 for the characteristics of included studies.

### Quality assessments

Seventeen studies were rated as high quality [19, 21, 24–26, 29, 33–36, 38, 41, 44, 45, 47, 50, 51], 11 as medium quality [23, 27, 28, 31, 37, 40, 42, 43, 46, 48, 49] while 3 studies [22, 30, 39] were of low quality. Please see Table 1 for the quality assessment of included studies.

**Table 1:** Quality assessment of included studies

Study number	Author (year)	Abstract and title	Intro and aims	Method and data	Sampling	Data analysis	Ethics and bias	Results	Transferability	Implications and usefulness	Total quality assessment score
1	Abrahamsson (2005) [19]	Good	Good	Fair	Fair	Good	Fair	Good	Fair	Good	32
2	DeWilde (2015) [27]	Fair	Fair	Poor	Fair	Fair	Fair	Fair	Poor	Fair	25
3	Everett (2005) [28]	Fair	Good	Fair	Fair	Fair	Fair	Fair	Fair	Fair	28
4	Longman (2018) [29]	Good	Good	Good	Good	Good	Poor	Good	Good	Good	34
5	Randall (2009) [30]	Fair	Very Poor	Poor	Poor	Poor	Good	Fair	Poor	Poor	21
6	Rezk-Hanna (2018) [47]	Good	Good	Good	Good	Good	Good	Good	Good	Good	36
7	Reardon (2016) [26]	Good	Good	Good	Good	Good	Good	Good	Poor	Good	34
8	Bull (2007) [22]	Poor	Fair	Poor	Fair	Fair	Good	Fair	Good	Fair	22
9	Colomar (2015) [48]	Good	Good	Fair	Poor	Fair	Fair	Good	Poor	Fair	28
10	Herberts (2012) [49]	Good	Good	Poor	Fair	Fair	Poor	Fair	Poor	Fair	26
11	Aquilino (2003) [23]	Good	Good	Fair	Fair	Poor	Fair	Fair	Fair	Fair	28
12	Thomson (2019) [25]	Good	Good	Good	Good	Good	Good	Good	Fair	Good	35
13	Thomson (2019) [21]	Good	Fair	Good	Good	Good	Fair	Good	Poor	Fair	31
14	Ashwin (2010) [31]	Fair	Fair	Fair	Fair	Fair	Good	Good	Fair	Fair	29
15	Bovill (2018) [33]	Good	Good	Good	Good	Fair	Good	Good	Good	Good	35
16	Bowker (2015) [34]	Good	Fair	Fair	Good	Good	Good	Good	Good	Fair	33
17	Britton (2017) [35]	Good	Good	Good	Good	Good	Fair	Good	Fair	Good	34
18	Butterworth (2014) [36]	Fair	Good	Fair	Fair	Fair	Good	Good	Fair	Fair	30
19	Gamble (2015) [37]	Fair	Fair	Fair	Poor	Fair	Fair	Fair	Fair	Good	27
20	Goszczyńska (2016) [38]	Good	Good	Good	Good	Fair	Good	Good	Fair	Fair	33
21	Haslam (2001) [39]	Poor	Fair	Fair	Poor	Poor	Poor	Fair	Poor	Fair	22
22	Haugland (1996) [42]	Fair	Good	Good	Fair	Fair	Very Poor	Fair	Fair	Fair	27
23	Hotham (2002) [40]	Fair	Good	Fair	Fair	Fair	Fair	Poor	Fair	Poor	26
24	Howard (2013) [41]	Good	Good	Good	Good	Good	Good	Good	Fair	Good	35
25	Lendahls (2002) [43]	Fair	Fair	Fair	Fair	Fair	Fair	Good	Fair	Fair	28
26	Naughton (2013) [44]	Fair	Good	Good	Fair	Good	Fair	Good	Fair	Fair	31
27	Naughton (2018) [51]	Good	Fair	Good	Good	Good	Fair	Good	Fair	Good	33
28	Petersen (2009) [45]	Good	Good	Good	Good	Good	Fair	Good	Good	Good	35
29	Wiggington (2013) [46]	Good	Fair	Poor	Fair	Fair	Fair	Good	Fair	Fair	28
30	Wood (2008) [50]	Good	Good	Good	Fair	Fair	Fair	Good	Fair	Good	32
31	Bar-Zeev (2019) [24]	Good	Good	Good	Good	Good	Good	Good	Fair	Good	35
32	Reeks (2020) [20]	Good	Good	Good	Fair	Good	Fair	Good	Fair	Good	29

*Total Quality Assessment Score out of 36

*4 = Good; 3 = Fair; 2 = Poor; 1 = Very Poor. High quality: 30–36 points; medium quality: 24–29 points; low quality: 9–24 points*

### Qualitative COM-B analysis

Sub-headings of Capability, Opportunity and Motivation, and their subcomponents were used to either group results into smoking cessation care provided by HPs to PW or received by PW. Intervention functions, the range of functions within an intervention that support behaviour change and Policy categories from the BCW [14] (i.e., the middle (red) and outer (grey) rings) denoted as “IF” and “P” in brackets. Please see Table 2 for representative quotes related to each COM-B theme.

**Table 2:** Quotes related to each COM-B theme

COM-B Model	Quotes from Health Professional Perspectives	Quotes from Women’s Perspectives
**Physical capability**	*I always feel a bit concerned about doing actually more* *harm than good insofar as you know these women that appear* *to not be smoking very much.****HP- Bar-Zeev, 2019- 1st order***	*.*. *. I think it was a very minimal plan that had been laid out. I think there was two or three questions and that was it.* **Women- Bovill, 2018- 1st order**
**Psychological capability**	*Sometimes they have so many stressors in their life that they just don’t think they can give it (smoking) up…. A lot of pregnant women...would cut down on smoking, but if...their stress level rose...their smoking rose with their stress level.* ***HP- Aquilino, 2003- 1st order*** *I think that if the woman gets too much stressed about the fact that it is forbidden to smoke, then the only thing you can say, is: “Alright, you can smoke a few cigarettes a day.”* ***HP-Gynaecologist-De Wilde, 2015*- 1st order** *I advised this patient to see her GP for patches or gum, but her doctor told her that to use replacement products would be more dangerous than smoking so he said either keep smoking or do cold turkey so I don’t bother now****HP-Health Visitor, Bull, 2007- 1st order*** *I could use more information. There’s new stuff every day that relates to smoking, so I know there’s new and up-to-date stuff that we probably don’t know about.* ***HP- Aquilino, 2003- 1st order***	*I can remember the conversation we had about it and [the smoking cessation advisor] was letting me know where I can put [the patches] and what not, but to myself I just thought no, that’s just a bit too – you know you sit there thinking about it. I don’t know, it’s weird, I just think it’s too close to the baby to be having all that nicotine going in.* ***Woman- Bowker, 2015- 1st order*** *The doctor so scared me at the ante-natal clinic when she said that there were new studies showing that the baby flinches every time you puff the cigarette, that it is really painful (for the fetus). I didn’t think it was that serious. So every time I smoke and I feel the baby move, I think of this. That makes me stop smoking at once.* ***Woman- Hauglan, 1996-1st order*** *Her (HP’s) eyes were just locked on mine when she was telling me (referring to the harm to the baby from smoking) and I was just like. .. OK! I thought stop looking at me!* ***Woman- Gamble, 2015*** [37***,*** 20]***, − 1st order*** *You’re doing it for yourself. A pat on the back and someone telling you that you done really good is enough to make you feel good, you know.* ***Woman- Butterworth, 2013*** [36]***- 1st order*** *Another mother described how she had been asked if she was a smoker when she visited the clinic when her baby had a bad cough. When she confirmed that she smoked the doctor said: ‘Then quit’, but he never offered any support.****Women, Lendahls, 2002- 2nd order***
**Physical opportunity**	*I don’t have the time. I am not the kind of person who wants to spend half an hour to motivate smoking cessation.* ***HP-Gynaecologist, DeWilde, 2015- 1st order*** *Lack of attractive educational resources to distribute to pregnant smokers.****HP-Doctor, Everett, 2005 2nd order***	*The posters prompt you to ask your doctor and while in the waiting room, since you have nothing to do you can read.* ***Woman- Colomar, 2015-1st order***
**Social opportunity**	*Having established a high-quality relationship with their patients and being able to provide continuity of care were perceived as potentially enabling midwives to promote smoking cessation.* ***HP-Midwife, Herberts, 2012 [46]- 2nd order*** *They don’t just do it because they think ‘Oh let’s have a cigarette’ but it is usually because something has happened that is unpredictable and they cannot go up against that unless they have a good buddy system in place.* ***HP- Health visitor, Bull, 2007- 1st order***	*I think it’d be easier to quit smoking if you had something like an AA meeting but for smokers…I think if I had the urge to have a cigarette, and you could call somebody and say “Well, you know, I’m really stressed out right now and I really need to talk or I’m going to light up a cigarette.* ***Woman, Britton,2007*** [35]***- 1st order*** *I think hearing other people’s stories and how they cope with it is helpful.* **Woman, Bovill, 2018** [33]**- 1st order**
**Automatic motivation**	*If you have a patient who maybe smokes 40 cigarettes [a day], it is impossible that she will quit smoking.* ***HP- Colomar, 2015- 1st order*** *I say that the baby becomes smaller due to the lack of nourishment, that it has a smaller refrigerator, thinner arteries. If they still don’t get it I show them a pretty horrible picture.* ***HP- Midwife, Abrahamsson, 2005*** [19]***- 1st order***	Not addressed in the articles
**Reflective motivation**	*And if everything is actually uncomplicated and low risk, except for the fact that they’re smoking, yeah, I’d just – I don’t think it’s really our primary or part of our core job.* **HP- Obstetrician, Longman, 2018- 1st order** *I don’t have the time. I am not the kind of person who wants to spend half an hour to motivate smoking cessation. I think that I am too highly qualified. That’s not my job, I have too many other things to do. I want to refer them [smokers] to a specialist.****HP-Gynaecologist, DeWilde 2015- 1st order*** *It is a waste of resources to talk to women about how to stop smoking if they are not interested.****HP- Bull, 2007, 1st order***	Not addressed in the articles

‘1st order constructs’ are quotes from study participants, ‘2nd order’ constructs are themes and subthemes from the article

### Physical capability

HPs’ physical capabilities to provide smoking cessation advice and interventions in pregnant smokers are not relevant, as most HPs are physically capable of achieving this end. However, this category touches on PW’s physical capability to quit smoking, as many may experience physical dependence. PW stated that HP did not sufficiently assist them in dealing with their physical dependence [33, 45]. Because this concerns a HP’s psychological capability to aid PW in that way, we have placed it in that section.

### Psychological capability

This theme explored HPs’ psychological capabilities to provide effective quit smoking advice to their clients as well as how HPs addressed the psychological capabilities of their clients for quitting smoking.

HPs’ knowledge about providing smoking cessation interventions was a significant factor affecting the delivery of smoking cessation advice to their clients. HPs believed that greater knowledge about providing smoking cessation interventions might help them provide better smoking cessation care to PW who smoke (IF – education) [23, 49]. Lack of knowledge about providing smoking cessation interventions sometimes led to low self-efficacy in HPs to change the behavior of their pregnant clients who smoke [19–24, 27, 48]. This also supports the issue of HPs lack of knowledge about NRT added to the issue of HPs not being able to provide effective quite smoking advice.

Developing sensitivity towards PW’s psychological state was of paramount importance to the success of the smoking cessation intervention by the HPs and a known factor affecting the psychological capability of PW to quit smoking [41]. HPs acknowledged that PW, trying to quit smoking, might not be able to do so successfully due to socio-economic stress and resultant psychological challenges [23, 37, 50].

In such cases, some HPs utilized their psychological skills to help evoke feelings of control and empowerment in PW [28]. This may be achieved by providing practical stop smoking strategies that were easy to follow (IF - training) and hence more likely to be successful [19]. However, some HPs lacked training (IF -education and training) to address smoking in PW, especially with women who were stressed, had unsuccessful quit attempts or did not want to quit [23, 49, 25]. Stress was detrimental to PW, and in rare instances, HPs encouraged PW to continue smoking to avoid the stress attributed to quitting attempts [36, 38, 42, 51, 48]. Communicating the best health advice (IF - education) without making the PW feel guilty, while also trying to maintain a congenial relationship (IF -enablement) with their client, was often challenging for the HPs [19, 22, 24, 29, 30, 35, 42, 48, 52, 53]. Some HPs overcame this dilemma by recommending cutting down to reduce smoking-related harms rather than quitting, perhaps potentially preserving the relationship with their client (IF – enablement) [23, 24, 27, 29, 33, 40, 43, 46, 48, 53].

Although NRT is known to improve the physical capability of smokers to quit by reducing withdrawal symptoms, NRT was considered controversial by HPs who were reluctant to prescribe it or did not offer it frequently (IF - education) [22, 24, 27, 29, 33, 37]. Inadequate institutional policies and guidelines contributed to suboptimal use of NRT (e.g., not prescribing NRT to PW unless they have had a failed unassisted quit attempt) (P– guidelines) [22, 24, 27, 48]. Lack of knowledge among HPs affected the motivation of HPs to prescribe NRT, potentially contributing to continued smoking among PW [22, 29].

Skepticism about NRT was quite prevalent among PW [22, 31–34]. Widespread uncertainty about NRT among PW may indicate a need for a more detailed and comprehensive conversation during the consultation about NRT use to address doubts and concerns about the potential harms of NRT versus the benefits, although the HPs were not always successful in this endeavor (IF - enablement) [31–34].

HPs who had never smoked found it difficult to empathize with their pregnant clients who smoked [49]. HPs who themselves smoked hardly provided any smoking cessation counseling [52]. PW corroborated that HPs who smoked did not encourage them to quit or were not insistent enough [35, 52, 53]. For example, PW states this in McLeod, et al. [53]*: “My specialist he smokes so (he) does not advocate giving up smoking.”.*

In contrast, HPs who were ex-smokers sometimes acted as role models for the PW to quit smoking (IF – modeling): *“I tell them that I did it so they can jolly well do it too… because I’ve smoked”* McLeod, et al. [53]*.* HPs who themselves were ex-smokers were perceived as less judgmental (IF - enablement) and more understanding of PW’s smoking.

HPs reported PW becoming defensive if smoking cessation consultations evoked stigmatizing feelings of ignorance, guilt, and irresponsibility [24, 49, 50]. Although smoking cessation advice from HPs was expected and acceptable [43, 53, 54]. some PW resisted counselling when HPs initiated a dialogue about smoking and may counter HP advice with anecdotes and arguments that contested the smoking cessation narrative of HPs [27, 28, 35, 51, 45, 48, 50].

However, this does not necessarily prevent HP from deliberately using persuasion (IF - persuasion). Some PW supported HPs using ‘shock tactics’ to get the message of smoking cessation through to them [35, 42].

Some HPs considered providing smoking cessation advice to PW who were stressed to be risky in case their advice could have the opposite effect [38]. Conversely, some PW described their experiences of feeling pressured, stigmatized, and sometimes intimidated by the HPs for being a PW who smokes [35, 37]. Monetary compensation for quitting was generally not considered acceptable by both HPs and PW, as quitting smoking was considered to be linked to PW’s intrinsic value and self-worth rather than an extrinsic reward [36].

HP training in smoking cessation emerged as a distinct facilitator that increased the psychological capability of HPs to support smoking cessation in PW or refer them to other HPs who can offer smoking cessation advice [49]. Developing specific skills, such as motivational interviewing or how to discuss cessation while the PW are trying to quit, was especially desired [24, 29]. Some HPs implied that they are only able to provide very rudimentary counseling regarding smoking and its harmful effects on the fetus.

However, the lack of training opportunities was widespread [22, 29, 48, 52], and some HPs had not had any training at all [22, 29, 52]. These lacunae were specifically in the domains of asking, assessing, and advising about smoking cessation in a way that does not offend or alienate pregnant clients [29]. HPs wanted smoking cessation training to be ongoing and training content updated frequently to keep up with the new guidelines and research (IF – training; P – guidelines) [23, 48].

PW complained that some HPs often asked about their smoking status but never provided any practical support to help them quit pointing towards a lack of skills for providing holistic smoking cessation care [33, 40, 43, 49, 54].

### Physical opportunity

This theme described opportunities afforded to HPs by their physical environment for providing smoking cessation care to PW, and opportunities that PW reported were provided to them or were lacking from HP for smoking cessation care.

Some PW complained that they were not adequately informed about the harmful effects of smoking by their HPs, nor were encouraged on their attempts to cut down or quit smoking [46, 48, 49].

Time for HPs to provide smoking cessation support was limited, as were the resources to educate PW [22, 27, 35, 45, 48, 49, 52]. HPs (and sometimes PW) commonly felt they did not have enough time to provide adequate assistance to help PW quit, or that motivating PW to quit would be a waste of time (automatic motivation) [22–24, 27, 45, 48, 52].

HPs often integrated quit smoking discussions with physical examinations to save time. Providing group quit smoking information sessions (IF -environmental restructuring) was suggested as a way to save time. HPs suggested that the waiting time before the consultations could be used to show quit smoking videos to PW and their partners, saving time during the consultation (IF - environmental restructuring) and motivating PW to quit (IF = persuasion) [26, 48]. However, HPs went further to suggest that pressure (IF - coercion) may be used to force PW to watch and confirm viewing the content [35].

Counseling strategies, such as detailed and structured protocols, saved time and were desired by HPs [24, 50]. These resources could also enhance their psychological capability by increasing knowledge and confidence in delivering smoking cessation interventions. However, some HPs highlighted that there were no clear guidelines or pathways (P- guidelines) in their institution on providing smoking cessation assistance to PW [22, 27, 48].

In some instances, informational resources such as pamphlets, educational videos, and other self-help material were not readily available to HPs (despite PWs wanting to use them), making it difficult for them to provide smoking cessation health education specific to PW (P - communication/marketing) [23, 28, 48].

HPs and PW discussed different modes of delivering quit smoking advice. Face to face delivery was considered the most successful [36]. Electronic means of communication such as telephone and email were thought to have a role by sending reminders or encouragement in between face to face appointments, thus improving PW’s reflective motivation and psychological capability to quit (P– service provision) [36]. HPs believed that smoking cessation advice should not overly depend on the traditional medical model of health information provision but rather be conveyed creatively through a wide array of resources, which may be more persuasive and motivating (reflective motivation for PW) [22, 36]. Along with face to face smoking related discussions with their doctors, midwives and other office staff, PW were open to seeing brochures, posters and other health promotional material in the doctor’s office, which they thought provided additional information and motivated them to discuss smoking with their HP (physical opportunity; IF - environmental restructuring; P- communication) [43, 48, 54].

### Social opportunity

This theme explored interpersonal influences, social factors, and opportunities available to HPs for providing smoking cessation support to PW.

HPs realized that the baby’s birth could be a significant opportunity for smoking cessation as PW have innate instincts to protect the baby, and this could motivate PW to stop smoking. Hence it was considered important to start a dialogue about smoking even if HPs detected a reluctance to talk about smoking in their clients [22]. A supportive, trustful, and a respectful relationship with the PW was considered paramount to a successful quit smoking intervention by the HPs (IF - enablement) [19, 29, 30, 48, 49, 53].

HPs believed that it was necessary to reduce social stigma associated with smoking and make smoking cessation advice non-judgmental, empathetic, and sensitive to PW’s needs and their environmental contexts (IF – enablement) [22, 28, 30, 36, 41].

In some close-knit communities, such as among Aboriginal populations, PW preferred that smoking cessation advice be delivered by Elders rather than the HPs as the Elders were more familiar with the PW and their circumstances (P – service provision) [33].

Social and peer support were considered central to motivating PW to quit and stay quit, and the role of HPs in facilitating such support [19, 22, 31]. According to some participants (PW), HPs provided social support by continual encouragement to quit [36, 44, 46]. PW who stopped smoking while pregnant, often relapsed when the baby was born, mostly due to various life stressors [23, 53]. HPs stressed the importance of social support and continued health promotion for PW to quit smoking and stay quit [19, 31, 53].

Opportunities to deliver quit smoking advice in the form of group sessions emerged as a preferred method of promoting smoking cessation and improving motivation among PW [33, 35, 36]. Structured addiction support meetings along the lines of ‘Alcoholics Anonymous’ and buddy systems were also considered useful for providing social support during a quit attempt [22, 35, 36].

Some HPs advocated group smoking cessation interventions for PW early in their pregnancy stages to save time (physical opportunity) or while performing pre-natal consultations [36, 39, 20].

PW concurred that an experiential opportunity involving witnessing or hearing about other people’s lived experiences of the effects of smoking during pregnancy would encourage them to stop smoking [35].

### Automatic motivation

This theme explored the factors that affect HP’s innate emotional reactions, impulses, and inhibitions related to providing smoking cessation support to PW. Identification of vulnerabilities such as stress and depression in PW acted as a deterrent to the provision of smoking cessation care by the HPs [23, 36, 38]. HPs instinctively tried not to offend their clients and make them feel supported.

HPs additionally considered it important to improve the motivation and knowledge of PW about potential smoking-related harms to the baby as an integral objective of smoking cessation care [23]. To this end, some HPs presented or wanted to present gruesome imagery (IF - persuasion) to accentuate the automatic motivation of PW to quit smoking [19, 35]. Identification of PW’s motivation to quit and reinforcing it was considered more successful than introducing new motivations by the HPs [35].

Pregnant women being praised for efforts to quit smoking, not being judged for smoking, and not made to feel guilty about smoking by HPs (IF - enablement) were significant automatic motivators for the PW to continue trying to quit smoking [26, 36] However, this was not always achieved and sometimes PW felt that they were being pushed or nagged to quit smoking [37]. Reassurance from HPs appeared to pacify PW’s fears about attempting to quit smoking.

Assumptions about the clients not wanting to quit smoking and skepticism about their chances to succeed in becoming smoke-free often informed whether or not the HPs provided smoking cessation care [29, 48, 20].

Smoking cessation consultations were often labeled “difficult” [49] HPs feared evoking pain, guilt, and shame in PW by discussing their smoking, and hence, the topic of smoking cessation [24, 45, 49]. HPs may instinctively avoid the issue altogether.

### Reflective motivation

This theme explored the processes and beliefs of HPs that promoted provision of smoking cessation care for their patients. Some health providers were intrinsically motivated to provide smoking cessation counseling and considered it their duty to assist PW to quit smoking [24, 26, 52]. On the other hand, others felt that it was not their role to provide smoking cessation interventions to their clients [29].

Role confusion affected HPs motivation to provide adequate smoking cessation. Clinicians reported they were not responsible for addressing smoking cessation with PW and it was better suited to come from smoking cessation specialists, GPs or midwives during pre-natal clinics [29, 55]. However, midwives described situations where the topic of smoking cessation was avoided, or they did not perceive it to be their responsibility either [27].

Several HPs expressed that they only considered providing smoking cessation assistance to PW if, during the consultation, they inferred that the PW wanted to quit smoking [22, 52].

HPs believed that success of a smoking cessation intervention depended on the inherent motivation of their clients to quit: *“if they don’t want to give up you’re bashing your head against a brick wall”* [29]. HPs who provided information about the harmful effects of smoking and quitting methods to PW patently empowered them with the knowledge to motivate an effective quit attempt.

## Discussion

This systematic review explored qualitative research from 32 studies (spanning more than 20 years) across the world about the reported experiences of PW and HPs during smoking cessation care. The analysis of the qualitative themes used the COM-B behavior change model’s Behaviour Change Wheel [15]. This model has been previously used to understand different behaviors such as NRT use [56], barriers and facilitators of health behaviors [57, 58] and uptake of health services [59]. Almost all studies included in this review were rated as high to medium quality, thus indicating greater reliability of the results.

This review found that there was a lack of key intervention functions and policies that could support health professionals provide good smoking cessation care. Smoking cessation interventions aimed at improving the physical capabilities of PW who smoke (i.e., to aid PW in dealing with the physical addiction to nicotine), were rarely described or addressed. Nicotine metabolism increases significantly during pregnancy [60], making nicotine withdrawal much more pronounced. Thus, HPs who are unable to improve the physical capability of PW through offering NRT may not be successful in enabling smoking cessation among their clients [61, 62]. Some PW may be wary of using NRT due to fears of nicotine harming their baby and thus prefer to continue smoking, or quit without pharmacological support. This lack of enthusiasm by both parties towards using NRT has been demonstrated in other reviews [63] and could significantly hamper the physical capability of PW to overcome their nicotine withdrawal symptoms and quit smoking successfully.

HPs may hold paternalistic beliefs that expecting PW who smoke to quit was too challenging for them. Literature suggests that HPs who are not confident of their counseling techniques will often adopt paternalistic approaches as opposed to HPs who take a patient-centered approach and act as friends and carers to their clients [64]. Stress among PW and lack of skills among HPs to manage it may promote HPs to recommend ‘cutting’ down rather than complete abstinence. Ingall et al. (2010) [65] similarly found that HPs who were smokers may support PW to cut down rather than quit completely. Despite some clients perceiving a recommendation to cut down as supportive [66], the latest evidence and smoking cessation guidelines conclude that complete abstinence is the best practice [67]. Potential benefits from smoking reduction are highly questionable [68]. Our previous research suggests that ‘cutting-down’ to assist towards cessation may delay PW from getting the benefits for their baby that could be achieved by complete abstinence [33]. A less prescriptive and more patient-centered approach may be more useful for behavior change [69].

Lack of time, training, and resources along with role ambivalence among HPs contribute to many missed opportunities to counsel PW about smoking cessation. Negative attitudes, lack of knowledge resulting in limited confidence to discuss smoking cessation, and perceptions of the topic as unpleasant and intrusive, are common barriers to the provision of smoking cessation care to PW who smoke. These factors combine to rob HPs of a significant social opportunity to address smoking among their pregnant clients [70]. Pregnant women who smoke are not consistently offered health information related to smoking and its adverse effects, smoking cessation advice, nor provided referrals [24, 71, 72]. Lack of opportunities for training in smoking cessation has been raised by other authors, similarly to our study [13, 73].

The results of our review align with themes raised by systematic reviews by Baxter, Everson-Hock, Messina, Guillaume, Burrows and Goyder [74] (23 papers) and Bauld, et al. [63] (9 studies) on perspectives of HPs on the delivery of smoking cessation care to PW. Baxter, et al. [74] highlighted HPs’ lack of interpersonal and counseling skills and skepticism towards their effectiveness in providing smoking cessation care, although unlike the present review, there was only limited reference in the Baxter’s review about NRT. Our findings confirm the results of Bauld, et al. [63] who found that there was a need to improve smoking cessation knowledge and confidence among HPs. Best strategies to support women identified in Bauld, et al. [63] review were routine CO screening, behavioral support, and access to pharmacotherapy. Pregnant women can under-report their smoking behaviours [75]. CO screening could better identify smoking behaviours among pregnant women allowing HP to provide better SCC support. In contrast to Baxter, et al. [74], the present review also examined the perspectives of PW about the smoking cessation care provided to them by HPs. Analysis of both perspectives through the lens of BCW can inform more effective policies and interventions.

### Strengths and weaknesses

All studies that fit the selection criteria were included in the review irrespective of quality making it the most far-reaching review of HPs’ and PW’s perspectives on smoking cessation care given and received during pregnancy. A strength of this study is that it advanced the use of the COM-B in a unique way by juxtaposing HP and women’s voices within the categories of the BCW. This review simultaneously analyzed both the perspectives of PW about receiving HP smoking cessation care and the HPs providing smoking cessation care to get a complete picture of the interaction between the PW and their HPs, the differences, and similarities. We only included studies published in English, and hence, the generalization of results to a broader range of non-English speaking countries is limited. This was also a global systematic review and hence local and cultural contexts have not been studied in detail. The main reason for this is majority of the studies were conducted in high-income countries which have similar health systems. Moreover, our aim was to look for commonalities across the globe as opposed to determining which attitudes were more prevalent in different countries.

### Implications for practice, policy, and research

There is a need to improve the knowledge and capabilities of HPs to provide effective smoking cessation care to their pregnant clients. This review could guide interventions to improve HPs capability and motivation to deliver smoking cessation interventions to PW along with encouraging policies to improve opportunities for HPs to deliver these interventions. This would include making available to HPs ongoing training opportunities, supplemented with clear guidelines, and effective resources. Secondly, smoking cessation training should include effective techniques to manage the clients’ stress and other negative mental effects, since stress and depression in PW are major deterrents to the provision of smoking cessation care [76]. Training could address HPs’ concerns that smoking cessation counseling will adversely impact their relationship with their pregnant clients, along with boosting their confidence to address smoking cessation care. Thirdly, policy gaps were identified by health professionals. Communication and guideline policies in health institutions and services, in particular, formulating smoking cessation policy with staff would improve SCC outcomes. Asking about smoking cessation status and the provision of smoking cessation advice should be normalized throughout the service and mandatorily undertaken by all HPs so that there is no role confusion about smoking intervention delivery. Fourthly, since many HPs hesitate to suggest NRT for quitting due to limited research in this field, well designed randomized controlled trials are required to strengthen the evidence base with regards to effectiveness and safety of higher doses of NRT in pregnancy [77].

## Conclusion

This study provided an in-depth analysis of smoking cessation care offered by HPs to PW who smoke. The results of this study are useful in identifying barriers to delivery of smoking cessation care by HPs as well as designing effective smoking cessation interventions that are most likely to be accepted by PW and implemented by HPs.

## Supplementary Information

**Additional file 1.** Search terms for literature review 31/07/2015.

**Additional file 2.** The characteristics of included studies. This of included studies including study first author and year, country, study focus and number of participants, study aim(s) and summary of results.

## Data Availability

Not applicable.

## Abbreviations

HP: Health Provider
PW: Pregnant woman/women-
SCC: Smoking Cessation Care
COM-B: Capability, Opportunity, Motivation, Behaviour
NRT: Nicotine Replacement Therapy
5As: Ask, Assess, Advise, Assist and Arrange
ABC: Ask, Brief advice and Cessation support
BCW: Behaviour Change Wheel
IF: Intervention Function
P: Policy categories
PRISMA: Preferred reporting items for systematic reviews and meta-analyses
MESH: Keywords and Medical Subject Headings (MESH)
