# Correction to: Health providers’ and pregnant women’s perspectives about smoking cessation support: a COM-B analysis of a global systematic review of qualitative studies

**DOI:** 10.1186/s12884-021-04094-9

**Published:** 2021-09-08

**Authors:** Ratika Kumar, Leah Stevenson, Judith Jobling, Yael Bar-Zeev, Parivash Eftekhari, Gillian S. Gould

**Affiliations:** 1 School of Medicine and Public Health, The University of Newcastle, University Dr, Callaghan, New South Wales 2308 Australia; 2 Braun School of Public Health and Community Medicine Hebrew University - Hadassah Medical Center, PO Box 12272, 91120 Jerusalem, Israel

**Correction to: BMC Pregnancy Childbirth 21, 550 (2021)**

**https://doi.org/10.1186/s12884-021-03773-x**

Following publication of the original article [1], the authors reported a couple of minor changes needed in the Abstract and Results section, including reference citations.

- In the result section of Abstract, “twelve on HP’s perspectives, 16 on PW’s perspectives” was amended to “13 on HP’s perspectives, 15 on PW’s perspectives”
- In the Results section of the article, the first line has been referenced. “Twelve focused exclusively on the HPs’ perspectives [19–30], 16 exclusively on PW’s perspectives [31-46]” has been changed to “13 focused exclusively on the HPs’ perspectives [19-30, 51], 15 exclusively on PW’s perspectives [31, 33-46]”
- Under Quality assessment “11 as medium quality [23, 27, 28, 31, 37, 40, 42, 43, 46, 48, 49]” has been changed to “12 as medium quality [20, 23, 27, 28, 31, 37, 40, 42, 43, 46, 48, 49]”.
- In the reference list, author names ‘Michie SAL, West R’ were amended to ‘Michie S, Atkins L, West R’.

The original article has been corrected.

## References

[1] Kumar R, Stevenson L, Jobling J, Bar-Zeev Y, Eftekhari P, Gould GS (2021). Health providers’ and pregnant women’s perspectives about smoking cessation support: a COM-B analysis of a global systematic review of qualitative studies. BMC Pregnancy Childbirth.

