# Supplementary material for: Health providers’ and pregnant women’s perspectives about smoking cessation support: a COM-B analysis of a global systematic review of qualitative studies
Source: BMC Pregnancy Childbirth. 2021 Aug 12;21:550. doi: 10.1186/s12884-021-03773-x (PMC8359058; doi:10.1186/s12884-021-03773-x)
Supplement: Supplementary file 1 — Additional file 1. Search terms for literature review 31/07/2015. [file 12884_2021_3773_MOESM1_ESM.docx]

Supplementary file 1: Search terms for literature review 31/07/2015

| Search number | Search term | Combined with OR |
| --- | --- | --- |
| 1 | Tobacco | OR |
| 2 | Smoking | OR |
| 3 | Smoking Cessation | OR |
| 4 | Tobacco use disorder | OR |
| 5 | Nicotine dependence | OR |
| 6 | Tobacco dependence treatment | OR |
| 7 | “Maternal tobacco smoking” | OR |
| 8 | Smok* | OR |
| 9 | Smoking treatment | OR |
| 10 | *1-9* | OR |
| 11 | Pregnancy | OR |
| 12 | Preg* | OR |
| 13 | Maternal behaviour | OR |
| 14 | Maternal | OR |
| 15 | Mother | OR |
| 16 | Perinatal Care | OR |
| 17 | Prenatal Care | OR |
| 18 | Antenatal | OR |
| 19 | *11-18* | OR |
| 20 | Health professional | OR |
| 21 | General practitioner | OR |
| 22 | Physician | OR |
| 23 | Family Practice | OR |
| 24 | Allied health personnel | OR |
| 25 | Doctor | OR |
| 26 | Specialist | OR |
| 27 | Medical practitioner | OR |
| 28 | Health personnel | OR |
| 29 | Midwife | OR |
| 30 | Gynecology | OR |
| 31 | Obstetrics | OR |
| 32 | Clinician | OR |
| 33 | Dentist | OR |
| 34 | Pharmacist | OR |
| 35 | Consultant | OR |
| 36 | *20-35* | OR |
| 37 | Attitude | OR |
| 38 | Skill | OR |
| 39 | Knowledge | OR |
| 40 | Perception | OR |
| 41 | Practice | OR |
| 42 | Belief | OR |
| 43 | Capacity | OR |
| 44 | Capability | OR |
| 45 | Confidence | OR |
| 46 | Priority | OR |
| 47 | Barrier | OR |
| 48 | Health Knowledge, Attitudes, Practice | OR |
| 49 | Attitude of Health Personnel | OR |
| 50 | Ability | OR |
| 51 | *37-50* | OR |
| 52 | *10, 19, 36,51* | AND |

Notes:

Note all search terms were ‘exploded’ (meaning the terms underneath these keywords were also searched for).

E.g. smoking includes the following list of terms:

·         cigar smoking

·         cigarette smoking

·         hookah smoking

·         pipe smoking

·         smoking

·         smoking, cigar

·         smoking, cigarette

·         smoking, hookah

·         smoking, pipe

·         smoking, tobacco

·         smoking, waterpipe

·         tobacco smoking

·         waterpipe smoking

Where possible, terms were matched to Mesh or database specific subject headings as well as used as a keyword.  E.g. Smoking/ as the subject heading and smoking.mp as the keyword in the database Medline.
