# Supplementary material for: Health providers’ and pregnant women’s perspectives about smoking cessation support: a COM-B analysis of a global systematic review of qualitative studies
Source: BMC Pregnancy Childbirth. 2021 Aug 12;21:550. doi: 10.1186/s12884-021-03773-x (PMC8359058; doi:10.1186/s12884-021-03773-x)
Supplement: Supplementary file 2 — Additional file 2. The characteristics of included studies. This of included studies including study first author and year, country, study focus and number of participants, study aim(s) and summary of results. [file 12884_2021_3773_MOESM2_ESM.docx]

Supplementary file 2: The characteristics of included studies

| **Study Number** | **First Author (year)** | **Country** | **Study Focus & Participant Numbers** | **Study Aim(s)** | **Summary of results** |
| --- | --- | --- | --- | --- | --- |
| 1 | Abrahamsson (2005) | Sweden | Health Providers N=24 (midwives) | To qualitatively describe the different ways in which midwives make sense of how to approach women smokers. | Four different story types of how the midwives made sense of their experiences in addressing smoking in pregnancy were identified: avoiding, informing, friend-making, co-operating. |
| 2 | DeWilde (2015) | Belgium | Health Providers N=17 (midwives, gynecologists) | To explore knowledge, beliefs, and practice among midwives and gynecologists concerning a smoking cessation policy for pregnant women and their partners. | Major themes reported were: basic knowledge regarding fetal and maternal risks associated with smoking during pregnancy; speciﬁc knowledge regarding national smoking cessation guidelines and the use of nicotine replacement therapy (NRT); the image of "the smoking pregnant woman"; the 5 A's framework; and Perceived need for smoking cessation training. "Ask" and "Advise" were part of a standard prenatal consultation; the next three steps were rarely implemented. Participants had a negative image of "the smoking pregnant woman": a low educated woman with a smoking partner and "bad examples" in their history. Barriers were fear of provoking resistance and lack of time and communication skills regarding smoking cessation. |
| 3 | Everett (2005) | South Africa | Health Providers N=15 (doctors) | To investigate the current smoking cessation practices and attitudes of doctors working in the public antenatal services as well as their perceived barriers to addressing the issue in the context of routine care. | The doctors in this study regarded HIV, poor nutrition, alcohol abuse, and psychosocial stress as equal or higher risks to pregnant women than smoking. They were unaware of counseling guidelines and pessimistic that they could influence the smoking behavior of disadvantaged pregnant women. Perceived barriers to interventions included: lack of counseling skills/ educational resources, other priorities, too little time, and the levels of stress currently experienced by doctors and midwives working in public sector hospitals. |
| 4 | Longman (2017) | Australia | Health Providers N= 27 (maternity service managers, obstetricians, gynecologists) | To explore the enablers and barriers to implementation of the Australian smoking cessation in pregnancy guidelines. | Participants confirmed that the implementation of smoking cessation guidelines was sub-optimal. Key barriers included systems that did not monitor, lack of knowledge about NRT, nature of addiction and barriers to quitting, skills and training, perceived time restrictions, 'difficult conversations', and perceiving smoking as a social activity. Enablers included clinicians' knowledge of the harms of smoking in pregnancy, communication skills, positive emotions, professional role and identity, the potential of training, and systems that regulated behavior. |
| 5 | Randall (2009) | United Kingdom | Health Providers N=7 (midwives) | To explore midwives' attitudes towards smoking and smoking cessation in pregnant women. | Four themes emerged: relationships; the context of smoking; competing issues; and competence in smoking cessation. The theme of competing issues shows an aspect of the phenomenon not previously seen in midwifery or nursing literature. |
| 6 | Reardon (2016) | United Kingdom | Health Providers N=8 | To explore widwives' experiences discussing smoking cessation with pregnant women during antenatal visits | Midwives overwhelmingly agreed it was their role to discuss smoking cessation with pregnant women during antenatal visits. Midwives deemed it important to take a non-judgmental approach however, expressed frustration when women seemed not to be concerned even when their unborn babies were at risk. |
| 7 | Rezkhanna (2018) | Central and Eastern Europe | Health Providers (nurses) & Pregnant Women N=81 | To describe the attitudes of nurses who are former and current smokers toward providing cessation interventions to patients as well as explore barriers and facilitators to their own quit eﬀorts when they were pregnant. | Nurses agreed that they should set a good example by not smoking; they should be involved in helping patients stop smoking, and needed additional training in tobacco control. Five common themes were identified as barriers to quitting: smoking cues in the environment, the presence of smokers in the environment, relapse postpartum, stress and nicotine addiction, and misperceptions about the dangers of smoking. Former smokers reported quitting after seeing the health consequences of smoking among their patients. |
| 8 | Bull (2007) | United Kingdom | Health Providers N= 23 (health visitors, midwives) | To explore the role of midwives and health visitors in the prevention of smoking during pregnancy and early parenthood. | The health visitors and midwives identified social and personal reasons why women smoke in pregnancy. Most felt a professional responsibility to intervene with smokers. However, they felt their intervention was ineffective unless patients were 'ready' to quit. They identified personal and organizational barriers to providing an effective service. Respondents held mixed views on the effectiveness of interventions for smoking cessation, highlighting the need for evidence-based training. Prescribing NRT was controversial, and health providers were cautious before considering prescribing it to pregnant women. |
| 9 | Colomar (2015) | Argentina and Uruguay | Health Providers N= 46 (obstetrician-gynecologists, midwives, nurses) & Pregnant Women N= 24 (ethnicity not reported) | To improve the understanding of systemic and individual factors influencing the implementation of the 5A's in prenatal care settings among prenatal clinic directors and providers and acceptance of interventions among pregnant women in Argentina and Uruguay. | Three themes emerged: health professionals, health systems, and patients. HP barriers to cessation counseling included inadequate knowledge and motivation, perceived low self-efficacy, and concerns about inadequate time and large workload. They expressed interest in counseling script. Health system barriers included low prioritization of smoking cessation and a lack of clinic protocols to implement interventions. Pregnant smokers lacked information on the risks of prenatal smoking and the difficulty of smoking cessation. |
| 10 | Herberts (2012) | United Kingdom | Health Providers N=15 (midwives) & Women N=10 (white British, black Caribbean, black African, mixed white and black Caribbean) | To identify and juxtapose midwives' perceptions of providing stop-smoking advice and pregnant smokers' perceptions of stop-smoking services. | The perceptions of midwives regarding the provision of advice were related to the outcome of advice, the relationship with patients, personal experiences, attributes, perception of role, the impact of external factors, and aspects related to pregnant smokers and pregnancy. Pregnant smokers' perceived barriers and facilitators to approaching stop-smoking services were categorized into areas of smoking behavior, advice from health professionals (e.g., being unaware that NRT is offered and allowed during pregnancy), stop-smoking services, and negative perceptions of pregnant women who smoke. |
| 11 | Thomson (2019)  Reference 19 | United Kingdom | Health Providers (stop smoking practitioners, midwives with smoking cessation training and managers) N= 19 | To identify and explain health providers perceptions of the barriers and facilitators to NRT adherence as part of smoking cessation care among pregnant women. | Health providers believed the pregnant women had misinformation from friends and family about the risks of smoking or the use of NRT during pregnancy and this misinformation undermined their ability to deliver smoking cessation care. Misinformation included how to use NRT and unrealistic expectations on the effectiveness of NRT without other smoking cessation support. Health providers believed some pregnant women felt pressured to quit which made them question if they should be prescribing NRT. |
| 12 | Thomson (2019) Reference 20 | United Kingdom | Health Providers N= 11 (stop smoking practitioners, midwives with smoking cessation training and managers) | To identify beliefs and attitudes of smoking cessation professionals towards using NRT as part of smoking cessation care for pregnant women. | Health providers preferred the use of NRT as part smoking abstinence and would only continue to support NRT use if the patient had only had a brief smoking relapse. Organizational policy and the financial cost of NRT influenced health providers attitudes towards the management and supply of NRT to the pregnant women. Health providers did not feel they could use NRT by advocating its use as part of a smoking harm reduction strategy. |
| 13 | Aquilino (2003) | USA | Health Providers (WIC providers) N= 25 | To examine the perspective of women, infant, and child clinic providers on offering smoking cessation interventions for pregnant women. | Factors influencing the ability of WIC staff to provide a smoking cessation intervention for pregnant women included available time, clinic priorities, staff approaches to clients, and staff training. In addition, providers expressed concerns about educational materials for clients as well as additional client issues that prevented smoking cessation. The absence of mechanisms to track clinic outcomes related to smoking cessation was also noted. |
| 14 | Ashwin (2010) | United Kingdom | Pregnant Women  N= 10 (ethnicity not reported) | To explore women's views surrounding the use of NRT to aid smoking cessation, and to raise professional awareness of women's concerns regarding smoking and NRT in pregnancy. | The study provided insight into women's problems and achievements when using NRT. The two main themes that emerged from the study were that NRT was an important component in stopping smoking, but this was in conjunction with support given by the midwife advisor. Both themes appeared to be equally important to the women in aiding their attempts at smoking cessation. Also, the importance of tailored interventions should not be dismissed when aiding women in smoking cessation. |
| 15 | Bovill (2017) | Australia | Pregnant Women N= 20 (Aboriginal) | To gather Aboriginal women's stories of smoking and becoming pregnant to identify the barriers in accepting smoking cessation support during pregnancy. | Major themes identified for women accepting support were: ambivalence towards a need for support, health professional advice, reduction in smoking, and attitudes to NRT. Women reported being advised to cut down, rather than to quit; reducing consumption may be a barrier to accepting NRT. Women recommended enhanced clinical support and Aboriginal community engagement in cessation care. |
| 16 | Bowker (2018) | United Kingdom | Pregnant Women N= 14 (White, mixed British and Caribbean) | To understand the experience of pregnant women who use NRT but discontinue or do not use the medication as recommended. | There were four main themes identified; expectations of NRT, the experience of using NRT, safety concerns, and experience of using e-cigarettes. Some women intentionally used NRT to substitute a proportion of their cigarette intake and smoked alongside. Most women smoked while using NRT. Women who underutilized NRT did so as they experienced side effects, or were concerned that using NRT instead of smoking could increase their nicotine exposure and potential for increased nicotine dependence or fetal harm. Most women spoke about the use of e-cigarettes as a smoking cessation method, but only a few had experienced using them during pregnancy. |
| 17 | Britton (2017) | USA | Pregnant Women N= 45 (White, Asian) | To increase our understanding of the experiences of pregnant smokers and their providers. | Four common themes emerged in both the provider and the pregnant women groups: barriers to quitting, mixed messages, approaches and attitudes, and program modalities. These themes corroborate previous findings that cigarette smoking is used for stress relief, especially when pregnancy itself is a stressor, and that pregnant women may feel guilty but don't want to be nagged or preached to. |
| 18 | Butterworth (2014) | United Kingdom | Pregnant Women N= 19 (ethnicity not reported) | To consult smoking cessation services and report pregnant women's views of smoking cessation delivery and potential service developments. | The main themes included: (1) improving access to clear, sensitive information on smoking and pregnancy; (2) perceptions of existing services; (3) improving current services: the right delivery and the right person; and (4) encouraging participation of pregnant smokers. Women described their preferences regarding different modes of receiving quit smoking information as well as their views about various NRTs. |
| 19 | Gamble (2015) | Australia | Pregnant Women N= 6 (ethnicity not reported) | To explore experiences of smoking cessation interventions, perceptions of smoking cessation interventions efficacy, and views for improving smoking cessation interventions in pregnancy. | An over-arching theme of 'missed opportunities' and four inter-related sub-themes encapsulated the predominantly negative experiences of the intervention for the women. The women's interest in quitting was hindered by a didactic communication style employed by maternity care professionals. The participants' information and support needs were reported as being superficially managed by maternity care professionals or Quitline workers who provided care in routine ways. Women felt that Quitline workers varied considerably in their knowledge about NRT and speciﬁc pregnancy cessation options. |
| 19 | Goszcyska (2016) | Poland | Pregnant Women N= 47 (ethnicity not reported) | To research the subjective lay justiﬁcations expressed by smoking pregnant women to explain their use cigarettes during pregnancy and to determine a typology for these justiﬁcations. | Thirty-five subthemes of lay justifications assigned to two main themes were developed. The first main theme includes convictions that undermine the need to break the nicotine addiction and the positive aspects resulting from quitting. The second main theme comprises beliefs that question the possibility to abstain from smoking and exaggerate the barriers in the process of quitting (including perceived ineffectiveness of NRT in pregnancy). |
| 20 | Haslam (2001) | United Kingdom | Pregnant Women N= 40 (ethnicity not reported) | To examine the extent to which pregnant smokers are aware of the health risks, how they rationalize their smoking and the prompts they suggest encourage them to smoke. | Respondents specified four factors working against their quitting: other smokers, lack of will-power, dependence, and negative affect. While aware of the health risks, half said they were not worried about these risks, citing previous uncomplicated pregnancies experienced by themselves and others. Nearly all had partners who smoked, which for many, was a disincentive to quit. Respondents were asked what information they had received and what they thought would be useful. They were skeptical about the usefulness of leaflets but thought that videos might be effective. |
| 22 | Hauglan (1996) | Norway | Pregnant Women N= 33 (ethnicity not reported) | To obtain insight into pregnant smokers' experience of the information received from doctor and midwife at the anta-natal clinic. | Pregnant women lacking the motivation to stop smoking seemed to be most satisfied with ante-natal care. The women interviewed saw doctors and midwives as responsible for raising the subject of smoking, and blamed them for disinterest. The findings suggest that pregnant smokers may be classified into four categories ("it could have been worse", "self-delusion", "self-confident," and "rational") and that intervention should be tailored to meet each woman's perception of control over smoking behavior. |
| 23 | Hotham (2002) | Australia | Pregnant Women N= 36 (ethnicity not reported) | To explore barriers to `quitting' smoking for pregnant women, their attitudes to using nicotine patches, and their perceptions of care provider counseling. | A number of barriers to achieving smoking cessation were identified. Pregnancy-specific barriers included skepticism about smoking-related harms. Other barriers, such as addiction to nicotine and the smoking behavior of others, particularly partners, are generic. The latter is magnified in pregnancy by the heightened complexities of familial relationships. The potential use of nicotine patches was acceptable to most women, although some high-risk patients expressed doubts about safety and believed continued smoking was preferable. |
| 24 | Howard (2013) | United Kingdom | Pregnant Women N= 27 (white, black African, black Caribbean, mixed/other) | Investigate whether pregnant women with mental disorders: a) are less likely to accept referrals to smoking cessation services, b) are less likely to stop smoking by delivery, and c) differ in their experiences of smoking, smoking cessation and smoking cessation services compared with pregnant women without mental disorders. | Pregnant smokers with diagnosed mental disorders reported that they and health practitioners did not prioritize smoking advice because of concern about adversely impacting mental health. |
| 25 | Lendahls (2002) | Sweden | Pregnant Women N=24 (ethnicity not reported) | To identify the significant factors that influence women to stop/ not stop smoking during pregnancy and the postnatal period. | Women who still smoked at their first visit to the antenatal clinic often had an established smoking pattern. They had vague knowledge about the risks of smoking during pregnancy. All women interviewed stated that the midwife played an important role in their motivation to stop/reduce smoking during pregnancy. Many women, however, lacked the support from doctors, delivery and maternity ward staff and district nurses. Smoking cigarettes occasionally was preferred to using nicotine gum or patches. |
| 26 | Naughton (2013) | United Kingdom | Pregnant Women N= 20 (ethnicity not reported) | To explore the accounts of pregnant smokers and quitters to investigate the role of their smoking beliefs in influencing their cessation behavior and the relationships of these with psychosocial factors related to pregnancy and antenatal care. | The cognitive dissonance generated by the discrepancy between smoking-related prenatal harm beliefs and continued smoking was a strong motivator to quit. However, difficulty in quitting led many to resolve this dissonance by endorsing disengagement beliefs, which downplayed the threat of smoking. Four main types of disengagement beliefs were identified. The tendency to support disengagement beliefs appeared to be influenced by two factors: uncertainty about how smoking and nicotine (including NRT) caused fetal harm and reassurance from health professionals and others that reduced smoking was sufficient for harm minimization. |
| 27 | Naughton (2018) | United Kingdom | Health providers N= 48  (midwives, obstetricians, health visitors, GPs, pharmacists,  service commissioners, and Stop Smoking Service advisors and managers) | To better understand the health professional's  perspective on how interactions between women, health care professionals, and the  environment influence how smoking is managed | At the interpersonal level, protection of client-professional relationships often inhibited frank discussions about smoking, and weak inter-service relationships affected Stop Smoking Service referral motivation and quality. At the Individual level, professionals felt community midwives had primary responsibility for managing their patients smoking, although midwives felt under-skilled doing this. The demands of unrelated organizational initiatives influenced midwives' perceived priority for addressing smoking. |
| 28 | Petersen (2009) | Sweden | Pregnant Women N= 12 (mixed ancestry) | To explore barriers to and possibilities for interactive communication between midwives and pregnant women regarding smoking behavior during pregnancy. | The findings indicated low levels of transparency and trust in antenatal visits. Lack of trust was related to categories such as conflicting personal capabilities and socio-cultural and medical expectations, combined with a didactic approach from caregivers. The unworthy woman was identified as the core category of the interviews describing how women feel in their relationship with midwives. A theoretical model illustrates possibilities for change in relation to an ideal situation where a supportive caregiver, congruent expectations and capabilities result in women feeling visible. |
| 29 | Wiggington (2013) | Australia | Pregnant Women N= 11 (ethnicity not recorded, however a participant self-reported as Indigenous) | To examine the experiences of Australian women who are pregnant and smoke, and any stigma and any unintended consequences for their smoking. | Women's talk constructed medical and social pressures as two separate dimensions of stigma, which they accepted or resisted, or – at times – did both. They also used discursive strategies to negotiate their position as 'good mothers' despite the stigma and spoke about the need to manage the contexts in which they smoked. |
| 30 | Wood (2008) | Australia | Health Providers N= 10 (Aboriginal Health Workers) & Women N= 40 (Indigenous) | The investigate attitudes and experiences among Indigenous women regarding smoking during pregnancy. | Results showed that smoking cessation, even in pregnancy, was not a priority for most women, given the considerable social and economic pressures. Smoking was believed to reduce stress and to provide opportunities for relaxation. Pregnancy did not necessarily influence attitudes to cessation, though women's understanding of the consequences of smoking during pregnancy was low. The reduction of cigarette intake during pregnancy was an acceptable and positive behavior change. The AHWs saw their role to be primarily one of support and were conscious of the importance of maintaining positive relationships. As a result, they were often uncomfortable with raising the issue of smoking cessation with pregnant women. |
| 31 | Bar-Zeev (2019) | Australia | Health Providers N= 19 (General Practitioners) | To explore the General Practitioners perceptions towards smoking cessation support with pregnant women | General Practitioners showed a lack of knowledge and communication skills in delivering smoking cessation support to pregnant women GPs tend to focus on the health risk association with smoking during pregnancy and did not offer treatment options including NRT or Quitline. |
| 32 | Reeks (2020) | Australia | Health providers N= 15 (General practitioners) | Identify and describe the barriers and facilitators to General Practitioners use of the 5As smoking cessation support with pregnant women. | Some health providers were not aware of the 5As of smoking cessation but may have used some aspects of the 5As: Ask, Advice and Assist stages as part of their care. No other approaches were described by participants. Concerns about damaging rapport with pregnant women influenced if they were to offer smoking cessation support. A lack of time was also identified as a common reason smoking cessation care was not discussed with pregnant women or only provided some aspects of care. |

Legend: USA – United States of America; NRT – nicotine replacement therapy; AHW – Aboriginal Health Workers; HIV – human immunodeficiency virus; WIC – Nutrition Program for Women, Infants, and Children; Ethnicity as reported in each study
